# Oviposition traits generate extrinsic postzygotic isolation between two pine sawfly species

**DOI:** 10.1186/s12862-017-0872-8

**Published:** 2017-01-19

**Authors:** Emily E. Bendall, Kim L. Vertacnik, Catherine R. Linnen

**Affiliations:** Department of Biology, University of Kentucky, 204 TH Morgan Building, Lexington, KY 40506 USA

**Keywords:** Ecological speciation, Reproductive barriers, Host adaptation, Phytophagous insect, Diprionidae, *Neodiprion*

## Abstract

**Background:**

Although empirical data indicate that ecological speciation is prevalent in nature, the relative importance of different forms of reproductive isolation and the traits generating reproductive isolation remain unclear. To address these questions, we examined a pair of ecologically divergent pine-sawfly species: while *Neodiprion pinetum* specializes on a thin-needled pine (*Pinus strobus*), *N. lecontei* utilizes thicker-needled pines. We hypothesized that extrinsic postzygotic isolation is generated by oviposition traits. To test this hypothesis, we assayed ovipositor morphology, oviposition behavior, and host-dependent oviposition success in both species and in F1 and backcross females.

**Results:**

Compared to *N. lecontei*, *N. pinetum* females preferred *P. strobus* more strongly, had smaller ovipositors, and laid fewer eggs per needle. Additionally, we observed host- and trait-dependent reductions in oviposition success in F1 and backcross females. Hybrid females that had *pinetum*-like host preference (*P. strobus*) and *lecontei*-like oviposition traits (morphology and egg pattern) fared especially poorly.

**Conclusions:**

Together, these data indicate that maladaptive combinations of oviposition traits in hybrids contribute to extrinsic postzygotic isolation between *N. lecontei* and *N. pinetum*, suggesting that oviposition traits may be an important driver of divergence in phytophagous insects.

**Electronic supplementary material:**

The online version of this article (doi:10.1186/s12862-017-0872-8) contains supplementary material, which is available to authorized users.

## Background

Evolutionary biologists have long recognized that natural selection plays an important role in the formation of new species [1–4]. However, it is only within the last two decades that ecological speciation—the process by which environmentally based divergent selection gives rise to reproductive isolation [5, 6]—has become the focus of sustained research effort. During this time, laboratory and field studies in a wide range of organisms have demonstrated unequivocally that ecological speciation occurs in nature [7–10]. Moreover, comparative data suggest that ecological divergence plays a fundamental and taxonomically general role in driving speciation [11]. Nevertheless, while some aspects of ecological speciation are now fairly well understood, many major questions—including the relative importance of different forms of reproductive isolation (RI), and the types of traits that generate RI—remain unresolved [8].

Any form of RI can, in theory, contribute to ecological speciation so long as it arises as a consequence of divergent natural selection. However, one form of RI that may be especially important is extrinsic postzygotic isolation (hereafter, EPI), in which intermediacy or maladaptive combinations of traits in hybrids causes low fitness in both parental environments [7, 12–14]. EPI is thought to be a particularly common form of RI in ecological speciation because, so long as hybrids are intermediate and intermediate environments are lacking, it is a direct and automatic result of divergent selection [8]. As such, EPI should be among the earliest barriers to arise during ecological speciation [8, 15]. Additionally, when there is gene flow between diverging populations, EPI will lead to direct selection for assortative mating via reinforcement [4, 16]. However, although EPI is one of only two forms of RI that are unique to ecological speciation (the other being immigrant inviability, [17]), it is understudied relative to other forms of RI. One possible reason for the dearth of EPI studies is that it is challenging to distinguish between extrinsic (ecologically dependent) and intrinsic (due to genetic incompatibilities) sources of reduced hybrid fitness [14].

To date, three techniques have been proposed to distinguish between extrinsic and intrinsic sources of postzygotic isolation. The simplest of these is to compare the fitness of F1 hybrids in the wild to their fitness in a benign environment, in which the source of ecologically based selection has presumably been removed. If reduced hybrid fitness disappears in the “benign” habitat, this implies that the reduction was environmentally dependent [18]. The main limitation of this approach is that it does not control for stress-related expression of intrinsic hybrid incompatibilities [18]. A second, more rigorous approach is to rear backcrosses of F1s to both parental forms in both parental environments. EPI predicts that each backcross type will perform best in the parental habitat to which it is most genetically similar [13]. A final technique is to examine how specific hybrid traits impact fitness in parental habitats. This approach requires knowledge of the traits contributing to EPI and can be accomplished in one of two ways, the first of which is to experimentally manipulate parental individuals to resemble hybrids (modify-parental-phenotype) [7, 8]. Parents with the hybrid trait are expected to have reduced fitness in the parental environments if that trait is generating EPI. For many traits and organisms, however, these phenotypic modifications would be impractical, if not impossible. An alternative to direct modification is to take advantage of trait variation in F1 hybrids, F2, or backcross individuals and track how different trait values and combinations impact fitness in parental environments (e.g, [19, 20]).

One group of organisms that has featured prominently in empirical and theoretical studies of ecological speciation and EPI is plant-feeding insects. Several lines of evidence support the hypothesis that changes in host use are an important driver of ecological speciation in insects, including: (1) phylogenetic studies that show elevated rates of diversification among lineages of phytophagous insects compared to non-phytophagous insects [21–23], (2) comparative studies that demonstrate an association between changes in host use and speciation [11, 24, 25] (but see [26]), and (3) a growing list of empirical case studies that have confirmed key predictions of ecological speciation (reviewed in [8, 27–29]). However, while evidence supporting ecological speciation in insects is strong, the contribution of EPI remains unknown. In particular, although indirect evidence for host-related EPI exists for many taxa (reviewed in [29]), few direct tests exist (but see [20, 30–33]). Moreover, in most cases, the specific traits contributing to EPI have not been identified (but see [20]). Understanding the mechanistic basis of EPI is critical if we are to understand whether biases exist in the types of traits (e.g., morphological, physiological, behavioral) that contribute to reduced hybrid performance.

To investigate EPI—and the traits that produce it—we focus here on pine sawflies in the genus *Neodiprion* (Hymenoptera, Diprionidae), a Holarctic group of pine specialists that develop in intimate association with their host plants: adults mate on the host, females embed their eggs in the host tissue, and larvae complete their development on the host, spinning their cocoons on or beneath the host [34]. Population genomic data from a single species, *N. lecontei*, indicate that divergence in host use contributes to population differentiation [35], and comparative data from multiple species indicate that host-associated population differentiation occasionally progresses to speciation [25]. However, the mechanisms linking divergent host use to population differentiation and RI have not been identified.

To explore mechanistic links between host-use divergence and speciation in *Neodiprion*, we examined a pair of sister species that differ in host use, *N. pinetum* and *N. lecontei* [36, 37]. *N. pinetum* is a specialist that feeds on *Pinus strobus*, while *N. lecontei* feeds on a wider range of *Pinus* hosts, but generally avoids *P. strobus*. These species will mate under no-choice conditions in the lab and produce viable, fertile offspring (*personal observation*). Two lines of evidence indicate that they hybridize in the wild as well: (1) we have collected hybrids—which are identifiable via their intermediate larval coloration—at multiple field sites (*personal observation*), and (2) mitochondrial introgression has occurred between these two species [36]. Nevertheless, despite widespread sympatry and occasional hybridization, these two species remain morphologically, behaviorally, and genetically distinct. These observations suggest that there are postzygotic barriers to gene flow. Given that lab-reared hybrids are viable and fertile (*personal observation*), we hypothesize that postzygotic barriers between *N. lecontei* and *N. pinetum* are largely extrinsic in nature, stemming from their specialization on different *Pinus* hosts.

**Fig. 1:**
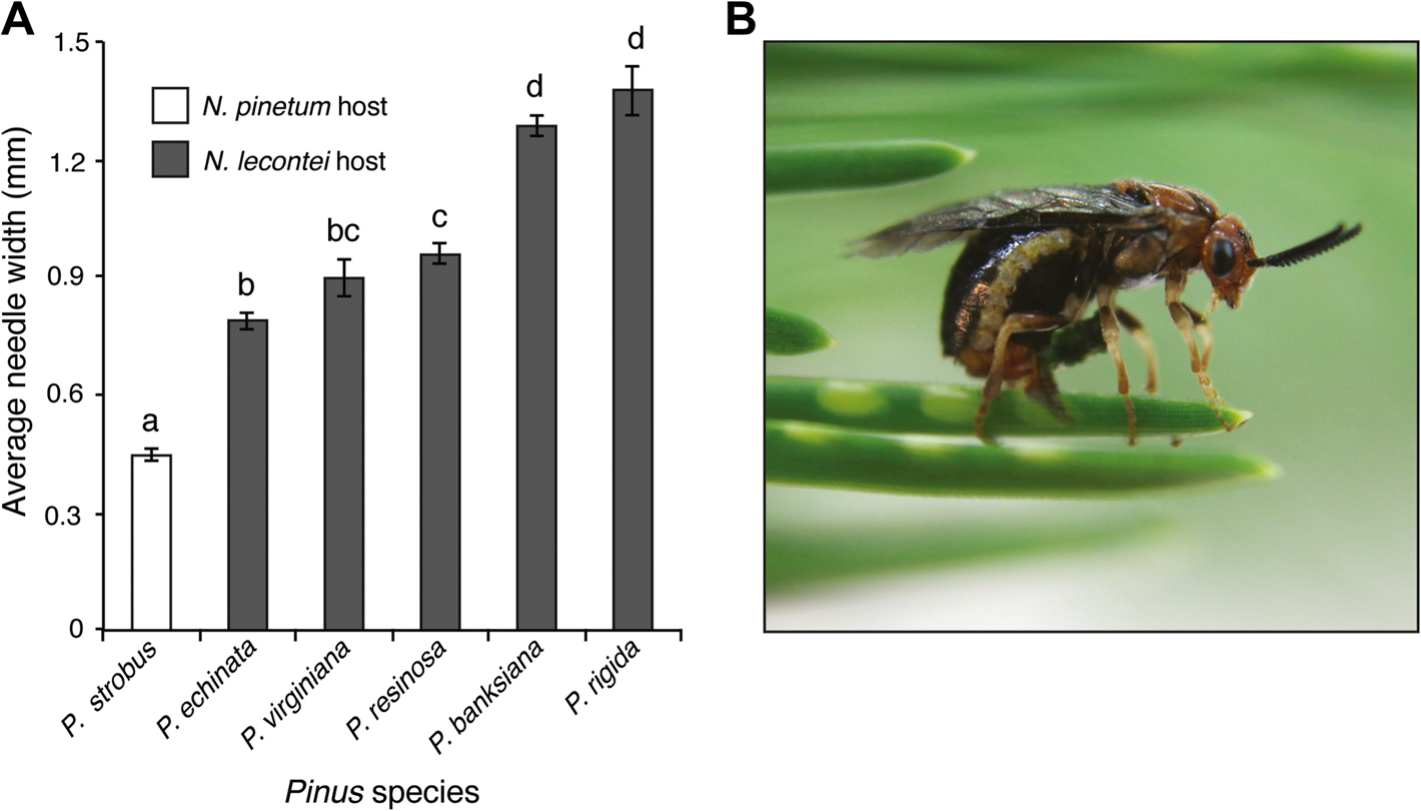
Needle width is a potential source of selection on oviposition traits. **a** Mean mature needle width (+/- SEM) of different pine species preferred by *N. pinetum* (*white*) and *N. lecontei* (*grey*). *Letters* indicate hosts that differ significantly at *P <* 0.05 (Additional file 1: Table S4). *N. pinetum*’s preferred host species has significantly thinner needles than those of *N. lecontei*’s hosts. **b** A *N. lecontei* female uses her saw-like ovipositor to carve an egg pocket into a pine needle (Photo by R.K. Bagley)

Additionally, we hypothesize that EPI has arisen as a consequence of divergence in oviposition traits. The most striking difference between the hosts of *N. pinetum* and *N. lecontei* is that *P. strobus* needles are far thinner and less resinous than other *Pinus* hosts [38] (Fig. 1a). This difference is important because *Neodiprion* females use a saw-like ovipositor to carve egg pockets into the pine needle (Fig. 1b). The eggs must survive within these pockets for anywhere between a week to 8 months [39, 40]. During this period, two major sources of egg mortality across the genus are desiccation and drowning in pine resin. For example, if an ovipositing female cuts her egg pockets too deeply, she can damage the vascular bundle within the host needle, causing the needle to dry out and the eggs to die [41–44]. Alternatively, for resinous host needles, failure to sufficiently drain host resins can result in egg drowning [41, 43]. Given the substantial fitness costs of improper oviposition, selection is expected to favor a close match between oviposition traits (morphology and behavior) and host plant needle characteristics (needle width and resin content). When two species with divergent oviposition phenotypes hybridize, hybrid females may have reduced fitness stemming from trait intermediacy or maladaptive combinations of oviposition traits.

There are several oviposition traits that vary among *Neodiprion* species that may be shaped by divergent natural selection stemming from host plant needle characteristics. First, there is interspecific variation in the number of eggs laid per needle: while some species lay a single egg per needle, others lay anywhere from 2 to 20 eggs per needle [42]. Whereas laying many eggs per needle may help diffuse host resin defenses and ensure that at least some eggs survive [41], increasing the number of eggs per needle also increases the risk of needle desiccation and egg death [44]. Second, *Neodiprion* species vary in their tendency to cut a “preslit” on egg-bearing needles. A preslit is a non-egg-bearing cut located near the base of the pine needle, proximal to the eggs. This cut disrupts the resin canal, allowing for resin to drain from the needle [44]. Like increasing egg number, the cutting of a preslit is associated with both increased oviposition success on resinous hosts [41] and increased desiccation risk [43]. Finally, ovipositor morphology is highly variable across the genus and ovipositor characters are the primary traits used in species identification [45]. Although the fitness consequences of different ovipositor shapes and sizes have not been studied, we hypothesize that different needle characteristics favor different ovipositor morphologies. For example, host plants with thin needles are likely to favor a reduction in ovipositor size.

To test the hypothesis that divergence in oviposition traits produces EPI between *N. pinetum* and *N. lecontei*, we evaluated a series of predictions. First, we predicted that *N. pinetum* and *N. lecontei* would have behavioral and morphological traits that are suited to the needle characteristics of their respective hosts. Specifically, we predicted that *N. pinetum* females would have oviposition traits that reduce the probability of needle desiccation (few eggs per needle, no pre-slits, and small ovipositors), while *N. lecontei* females would have oviposition traits that reduce the risk of drowning in host resin (many eggs per needle, pre-slits, and large ovipositors). Second, we predicted that hybrids and backcrosses would have reduced oviposition success (i.e., egg hatching) compared to each species, and that this reduction in success would be host dependent. Finally, if EPI is generated by oviposition traits, we predicted that oviposition success of backcrosses would be dependent on their oviposition traits. Together, our results provide compelling evidence that maladaptive combinations of oviposition traits contribute to extrinsic postzygotic isolation in *Neodiprion lecontei* and *Neodiprion pinetum*.

## Methods

### Insect collection and rearing

We collected *N. pinetum* and *N. lecontei* larvae throughout the eastern United States (Additional file 1: Table S1). We brought larvae back to the lab, transferred them to plastic boxes (32.4 cm × 17.8 cm × 15.2 cm) with mesh lids, and fed them pine foliage from their natal host species *ad libitum*. We collected cocoons as they were spun and stored them in individual gelatin capsules until adult emergence. We maintained all larvae and cocoons at 22 **°**C, 70% relative humidity, and an 18–6 h light-dark cycle [40, 46]. Upon emergence, live adults were stored at 4 **°**C until needed for crosses, morphological measurements, or behavioral assays. To propagate additional generations, we placed adult females and males into a mesh cage (35.6 cm × 35.6 cm × 61 cm) with seedlings of the pine species they were collected on. We allowed the adults to mate and the females to oviposit. After the eggs hatched, we reared larvae as described above.

### Host needle width

*N. pinetum* uses *Pinus strobus* (white pine) exclusively, while *N. lecontei* has eight primary pine hosts (*P. banksiana, P. resinosa, P. echinata, P. palustris, P. elliottii, P. rigida, P. taeda,* and *P. virginiana*) [34, 47, 48]. To characterize the oviposition environment, we measured the widths of needles collected from ten trees from each of six *Pinus* species, including *Pinus strobus* and five of *N. lecontei*’s primary hosts (*P. banksiana, P. resinosa, P. echinata, P. virginiana, P. rigida*). Host collection locations are indicated in Additional file 1: Table S2. For each pine tree, we measured the width of ten needles using digital calipers (Mitutoyo CD-6”PMX), then averaged these values to produce an average needle width per tree. Although we did not measure needles from three of *N. lecontei*’s primary hosts (*P. palustris, P. elliottii*, *P. taeda*), we note that all three species have needle widths greater than 1 mm [49], which exceeds *P. strobus* needle widths (see below).

We used *P. strobus* and *P. banksiana* (jack pine) seedlings as hosts for oviposition in all experiments (purchased from Itasca Greenhouse in Cohasset, MN and North Central Reforestation, Inc. in Evansville, MN). We used *P. banksiana* because *N. lecontei* larvae from different populations tend to perform well on this pine species (*personal observation*; [50]), and because *P. banksiana* seedlings are readily available from nurseries. To assess how seedling needles (experimental hosts) compare to needles from mature hosts (typical hosts in nature), we measured needle widths for *P. strobus* and *P. banksiana* seedlings using the same approach described above. To analyze the differences in host needle width among mature pine species, and between mature hosts and seedlings, we performed two ANOVAs with Tukeys post hoc tests.

Although we didn’t measure the resin content for the pine hosts we used in our experiments, variation in resin canal number among pines is well characterized in the literature. Pines in the subgenus *Strobus*, to which *P. strobus* belongs, have 2–3 resin canals per needle [38, 51]. In comparison, all of the hosts *N. lecontei* uses are in the subgenus *Pinus,* which has 2–12 resin canals per needle [38, 51]. Thus, on average, we expect *P. strobus* needles to contain less resin than the *Pinus* species that are typically utilized by *N. lecontei*.

### Oviposition behavior

Our hypothesis that divergent selection has shaped oviposition traits in *N. pinetum* and *N. lecontei* predicted that *N. pinetum* (thin-needle specialist) would have a stronger preference for *P. strobus* and would lay fewer eggs per needle than *N. lecontei* (thick-needle specialist). We also predicted that, compared to *N. lecontei*, *N. pinetum* would cut fewer “preslits,” [43]. We evaluated these predictions via a choice experiment. We first placed females in a clear 3.25-ounce deli cup with a single male until mating occurred. *Neodiprion*, like most hymenopterans, have arrhenotokous haplodiploidy, in which unfertilized eggs develop into haploid males [46, 52]. Although both mated and unmated females will oviposit readily (*personal observation*), we used mated females in our *N. lecontei/N. pinetum* oviposition assays as a means of propagating these lines. We then placed each mated female in a mesh cage (35.6 cm × 35.6 cm × 61 cm) with two *P. banksiana* seedlings and two *P. strobus* seedlings. We checked the cage daily until the female either oviposited or died. In nature, adult *Neodiprion* females have a very short life span (3–4 days) that is completely dedicated to reproduction [42, 47, 53]; likewise, in our choice cages, oviposition (or death) reliably occurred within 1–4 days (*personal observation*). For each female, we scored whether or not oviposition occurred. When oviposition occurred, we recorded host choice. Because *N. pinetum* and *N. lecontei* tend to cluster all of their eggs on a single branch terminus [48, 53], host choice is best described as a categorical trait with two possible outcomes: *P. banksiana* or *P. strobus*. We excluded three females that laid eggs on both hosts (representing 3.15% of the total sample).

To describe oviposition pattern, we counted the number of eggs, the number of egg bearing needles (EBN), and the number of EBN with preslits. We then used these data to calculate, for each female, the average number of eggs per EBN (number of eggs/number of EBN) and the proportion of EBN with preslits (number of EBN with preslits/total number of EBN). We then placed the egg-bearing seedling into an individual mesh sleeve cage (25.4 cm × 50.8 cm), and watered as needed until egg hatching occurred. To more fully describe oviposition pattern differences between the species, we also examined egg spacing, which may or may not influence hatching success on different hosts. To quantify egg spacing, we removed all EBN after hatching and imaged ten randomly selected EBN per seedling with a Canon EOS Rebel t3i camera equipped with an Achromat S 1.0X FWD 63 mm lens. Using these images, we measured the space between eggs in ImageJ [54] and, for each female, averaged egg spacing data across each needle. Sample sizes for each oviposition trait we scored are given in Additional file 1: Table S1.

To determine whether the two species differ in willingness to oviposit, we analyzed the proportion of *N. pinetum* or *N. lecontei* females that oviposited on any host with a generalized linear mixed effects model using a logit link factor, species as a fixed effect, and population (where each collecting location/host species combination was considered a separate population) as a random effect nested within species. To determine whether the two species differ in host preference, we used the same generalized linear mixed effects model to analyze the proportion of ovipositing females that chose *P. strobus*. To determine whether the two species differ in the average number of eggs per needle we used an ANOVA with species, natal host, and population as fixed factors. To determine whether the two species differ in average spacing between the eggs, we used an ANOVA. To determine whether the two species differ in the proportion of needles with preslits, we arcsine transformed the data and performed an ANOVA on the transformed data with species and population as fixed factors.

### Ovipositor morphology

In addition to behavior, we examined ovipositor morphology, with the prediction that, compared to *N. lecontei*, *N. pinetum* would have smaller ovipositors. To characterize ovipositor morphology, we used five females from each of five populations from each species (*N* = 25 females per species; Additional file 1: Table S1). We used females preserved at -80 **°**C from either the parental phenotyping experiments or that had been frozen upon emergence. To control for body size, we measured the length of the forewing from the anterior junction of the forewing with the body to the tip of the forewing. We then removed the ovipositor, and mounted a single lancet (inner saw) using an 80:20 permount:toluene solution. We photographed each mounted lancet at 5× magnification using a Zeiss DiscoveryV8 stereomicroscope with an Axiocam 105 color camera and ZEN lite 2012 software (Carl Zeiss Microscopy, LLC Thornwood, NY). Using this software, we measured the length from the top of the second annulus to the top of the penultimate annulus, and measured width at the second annulus. We then performed morphometric analysis, which allows us to test for shape differences while controlling for size of the ovipositor. For this analysis, we placed nine landmarks and 21 sliding landmarks on each ovipositor (see “Results”). We then examined ovipositor shape using Geomorph [55]. We applied a general procrustes alignment by minimizing binding energy. To determine whether the two species differed in ovipositor shape, we performed a procrustes ANOVA with forewing (as an allometric measurement), species, and population as fixed factors. To visualize the differences in ovipositor shape, we performed a principle components analysis in Geomorph. To determine whether the two species differed in ovipositor length or width, we used ANOVAs that included species, population nested within species, and forewing length as fixed factors. We completed all measurements and landmark placements in ImageJ version 1.49 V [54].

### Cross oviposition behavior and success

**Fig. 2:**
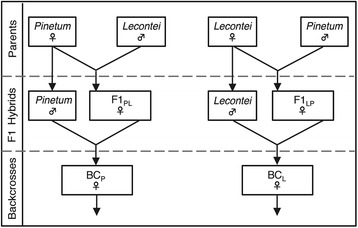
Cross design for assessing postzygotic isolation. Because *Neodiprion* have haplodiploid sex determination, unfertlilized eggs from an interspecific mating will produce male offspring of the mother’s genotype. Backcross females were unmated, while parental species and F1 hybrid females were mated

If postzygotic isolation contributes to reproductive isolation between *N. pinetum* and *N. lecontei*, hybrids should have reduced fitness relative to pure parental species; if this isolation is ecologically dependent, this reduction in fitness should be host-dependent. To test these predictions, we used the cross design outlined in Fig. 2 to generate F1 and backcross individuals between a *N. pinetum* population collected on *P. strobus* in Crossville, TN and a *N. lecontei* population collected on *P. echinata* (shortleaf pine) in Lexington, KY (Additional file 1: Table S1). Because *Neodiprion* are haplodiploid, males resulting from an interspecific cross carry maternal chromosomes only (Fig. 2). Our crosses involved six types of female, which we compared to make inferences regarding postzygotic isolation: parental *lecontei* (L), parental *pinetum* (P)*, lecontei* female-*pinetum* male F1 (F1_LP_), *pinetum* female-*lecontei* male F1 (F1_PL_), *lecontei* backcross (BC_L_), and *pinetum* backcross (BC_P_). Larvae were reared on the oviposition host that their mother chose. F1_PL_ females used in the cross were reared on *P. strobus* and F1_LP_ females were reared on *P. banksiana*. Backcross females were reared on a mixture of *P. strobus* and *P. banksiana.*

We placed individual females of each cross-type in a choice cage as described above (see “Oviposition behavior”) and recorded whether or not oviposition occurred and, when it did occur, the preferred host. The backcross females were unmated, while all other females were mated. As we are specifically interested in reduced fitness due to oviposition traits, we used oviposition success as our measure of female performance. A female was considered to have “successful” oviposition if at least one of her eggs hatched and “unsuccessful” oviposition if no eggs hatched within 4 weeks. We chose 4 weeks as a cut-off because this is well beyond the typical egg development time for both species under our rearing conditions (generally <16 days, *personal observation*).

Finally, we attempted to recover every female as soon as possible after death or oviposition occurred. Recovered females were preserved in 100% EtOH and stored at -20 °C for future use. Sample sizes are located in Additional file 1: Table S3.

To determine whether the direction of F1 cross (i.e., F1_LP_ vs. F1_PL_) differed in oviposition willingness, preference, or success, we used Z-tests. Because we did not observe any significant differences (see “Results”), we combined both cross-types into a single F1 category for the remaining analyses. To determine whether female cross-type (L, P, F1, BC_L_, BC_P_) differed in willingness to oviposit or in host preference, we used GLMs with a logit link factor and cross-type as a fixed effect, followed by post hoc Z-tests.

When there is postzygotic isolation, hybrids have reduced fitness compared to parental forms. To determine whether hybrids had reduced oviposition success compared to the parental species, we analyzed our hatch success data with a GLM using a logit link factor with cross-type as a fixed factor, followed by post hoc Z-tests. Additionally, if postzygotic isolation is “extrinsic” (due to the host plant), then oviposition success should be host-dependent. More specifically, each backcross type is expected to have the highest fitness (oviposition success) in the environment corresponding to the parent to which it is most similar genetically (i.e., there should be a cross-type-by-host interaction, Additional file 1: Figure S1) [13]. To test these predictions, we used the same GLM model as for total postzygotic isolation, but added the interaction between cross-type and chosen host to the model. Because both BC_L_ and BC_P_ females would have been reared on whatever host their F1 mother chose, it is possible that rearing host may have influenced oviposition success of backcross females. To control for a possible rearing host effect on oviposition success of backcross females, we added rearing host as a fixed effect to a GLM model that included backcross type (BC_L_ or BC_P_), oviposition host, and their interaction. We used Z-tests for all post hoc tests.

### Impact of oviposition traits on BC_L_ oviposition success

If oviposition traits are under selection and contribute to reduced hybrid fitness, the oviposition success of hybrid females should be dependent on these traits. To test these predictions, we focused on the BC_L_ females because they were the only cross-type for which we had an appreciable sample size for all relevant traits (ovipositors, oviposition pattern, and hatching success). Additionally, because there was very little variation in hatch success on *P. banksiana* (see “Results”), we focused our analyses on *P. strobus*, with the prediction that the BC_L_ females with *pinetum*-like traits (small ovipositor and few eggs per needle) would have the highest oviposition success on *P. strobus*. For these analyses, we scored oviposition success as a binary trait (hatch or no hatch) as described above. The results of the parental oviposition behavior assay indicated that *N. pinetum* females have a highly consistent and diagnostic oviposition pattern of three or fewer widely spaced eggs per needle (see below). To describe oviposition pattern, we therefore assigned each female to one of two categories: “*pinetum*,” if she laid three or fewer widely spaced eggs per needle and “non-*pinetum,*” if she laid more than three eggs per needle and/or eggs were spaced close together. To describe ovipositor morphology, we dissected and mounted female ovipositors as described above, with the addition of a rehydration step for EtOH-preserved females. The rehydration step consisted of six 10-min incubations of the female abdomen (at room temperature) in decreasing EtOH concentrations (100, 95, 80, 65, 50, and 25% EtOH), followed by overnight incubation in water.

To determine whether having a *pinetum*-like oviposition pattern increased the proportion of BC_L_ females whose eggs hatched on *P. strobus*, we used a GLM with a logit link factor and oviposition pattern as a fixed factor. Next, to determine whether “successful” females had more *pinetum*-like ovipositors than “unsuccessful” females, we used Geomorph (procrustes ANOVA accounting for forewing length) to compare ovipositor shape between females with and without egg hatching on *P. strobus* [55]. To determine whether ovipositor size affected the hatching rate on *P. strobus*, we performed separate GLMs with length and width data, both with a logit link factor and size as a fixed factor. To determine if oviposition pattern was correlated with ovipositor morphology we performed separate ANOVAs for ovipositor length and width, and a procrustes ANOVA for ovipositor shape.

Finally, we also used the BC_L_ data to determine whether there was any relationship between host choice and oviposition traits, which may occur if females exhibit behavioral plasticity (e.g., alter oviposition behavior based on chosen host or alter host preference based on having a particular ovipositor morphology). To determine whether host choice correlates with oviposition pattern we used a GLM with a logit link factor and chosen host as a fixed factor. To determine whether host choice correlates with ovipositor morphology, we performed a procrustes ANOVA. To determine if ovipositor length and width correlated with oviposition host we performed two ANOVAs. All statistical analyses were performed using R version 3.2.3 [56].

## Results

### Host needle width

Mature *P. strobus* had significantly thinner needles than all of the *N. lecontei* hosts (F_5,54_ = 72.42, *P* < 0.001, Additional file 1: Table S4, Fig. 1a). Likewise, *P. strobus* seedlings had significantly thinner needles than *P. banksiana* seedlings (*P* < 0.001, Additional file 1: Figure S2 and Table S5). However, because needles from *P. banksiana* seedlings were thinner than mature foliage (*P* < 0.001) and needles from *P. strobus* seedlings did not differ significantly from mature foliage (*P =* 0.15), the differences between our experimental hosts (F_3,36_ = 188.47, *P* < 0.001) are likely to be less extreme than differences typically experienced by ovipositing females in nature. In the discussion, we consider possible implications for the difference between seedling needles (experimental hosts) and mature needles (typical hosts).

### Oviposition behavior

**Fig. 3:**
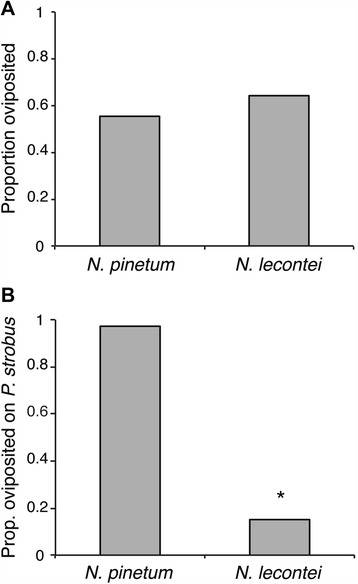
*N. pinetum* females preferred *P. strobus* more strongly than *N. lecontei* females*.* **a** *N. pinetum* and *N. lecontei* did not differ in the proportion of females that laid eggs when placed in a host choice arena (*P* > 0.05). **b** Of the females that oviposited, the proportion that chose *P. strobus* was higher for *N. pinetum* than *N. lecontei* (*P* < 0.05)

**Fig. 4:**
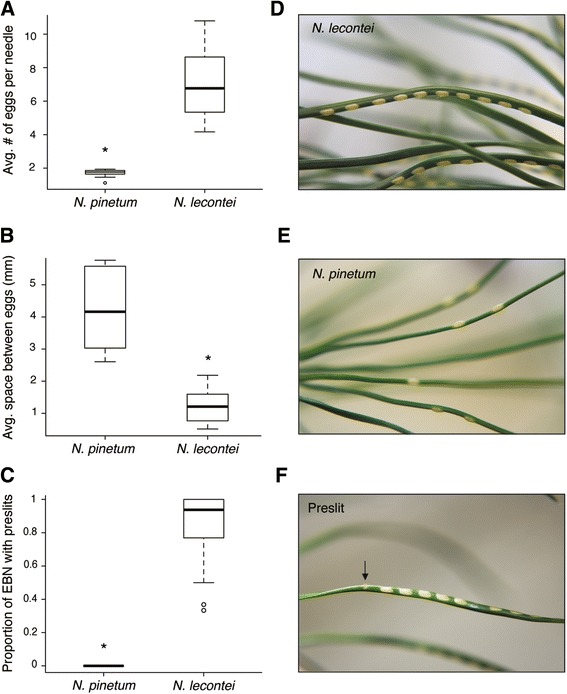
*N. pinetum* and *N. lecontei* females differed in their egg-laying pattern. **a** On average, *N. pinetum* females laid fewer eggs per needle than *N. lecontei* females. **b** On average, *N. pinetum* females spaced eggs farther apart than *N. lecontei* females. **c** Across all egg-bearing needles (EBN), *N. pinetum* females cut preslits less often than *N. lecontei* females. All comparisons were significant at *P* < 0.05. **d** Representative oviposition pattern of *N. lecontei* females: many, closely spaced eggs per needle. **e** Representative oviposition pattern of *N. pinetum* females: few, widely spaced eggs per needle. **f** A preslit (indicated by an *arrow*) cut by a *N. lecontei* female on a *P. banksiana* seedling (photos by R.K. Bagley)

*N. pinetum* and *N. lecontei* did not differ significantly in the proportion of females that oviposited (χ^2^_1_ = 0.14, *P* = 0.28, Fig. 3a). However, the two species did differ significantly in host preference, with *N. pinetum* exhibiting much stronger preference for *P. strobus* than *N. lecontei* (χ^2^_1_ = 6.47, *P* = 0.0011, Fig. 3b). *N. pinetum* also laid fewer eggs per needle (F_1,25_ = 21,50, *P* < 0.0001, Fig. 4a): whereas *N. pinetum* laid an average of 1.7 eggs per needle, *N. lecontei* averaged 7.2 eggs per needle. *N. pinetum* females also spaced their eggs farther apart than *N. lecontei* (F_1, 22_ = 62.86, *P* <0.001, Fig. 4b). Images representative of *N. pinetum* and *N. lecontei* oviposition pattern are shown in Fig. 4d, e. Finally, *N. pinetum* females cut fewer preslits than *N. lecontei* females (F_1, 17_ = 46.12, *P* <0.001, Fig. 4c, f): whereas none of the *N. pinetum* females we tested cut a preslit, all *N. lecontei* females cut at least one.

### Ovipositor morphology

**Fig. 5:**
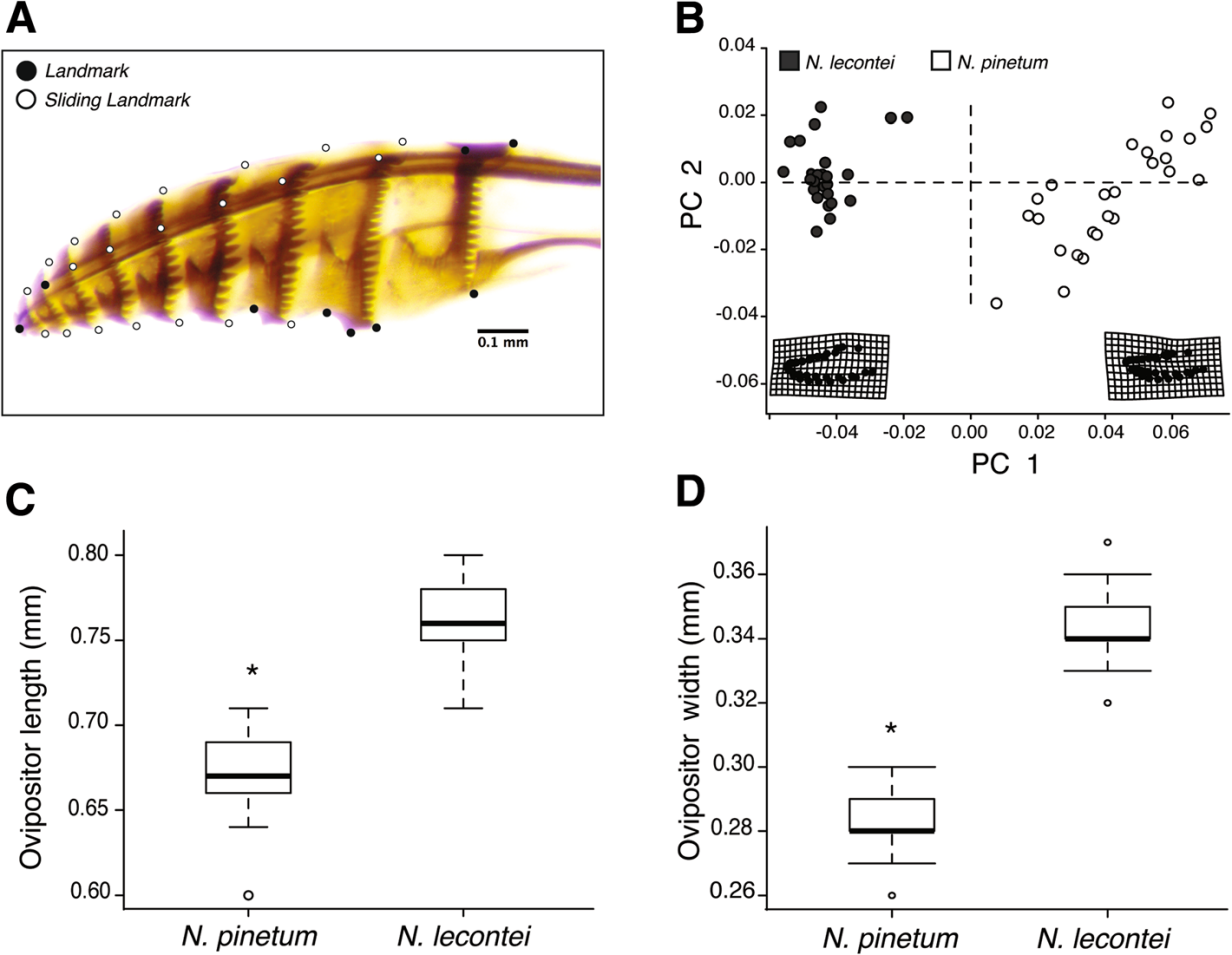
*N. pinetum* females had smaller, straighter ovipositors than *N. lecontei* females. **a** A representative *N. lecontei* ovipositor with landmarks (*black circles*) and sliding landmarks (*white circles*) used in morphometrics analyses. **b** Principle components analysis of ovipositor shape of *N. pinetum* females (*white circles*) and *N. lecontei* females (*grey circles*). The warp grids represent the change in ovipositor shape along principle component axis 1. **c** *N. pinetum* has narrower ovipositors than *N. lecontei*. **d** *N. pinetum* has shorter ovipositors than *N. lecontei*. Shape (**b**), length (**c**), and width (**d**) differences were all significant at *P* < 0.05

The 30 landmarks chosen for morphometric analysis are illustrated in Fig. 5a. *N. lecontei* and *N pinetum* females differed in ovipositor morphology: compared to *N. lecontei* ovipositors, *N. pinetum* ovipositors were shorter (χ^2^_1_ = 139.18, *P* <0.001, Fig. 5c), narrower (χ^2^_1_ = 186.71, *P* <0.001, Fig. 5d), and had a distinctly straighter shape (F_1, 39_ = 138.31, *P* < <0.001, Fig. 5b).

### Cross oviposition behavior and success

The direction of the F1 hybrid cross (i.e., F1_LP_ vs. F1_PL_) had no effect on the female’s willingness to oviposit (Z = 1.20, *P =* 0.23), preference (Z = 1.20, *P =* 0.23), or oviposition success (Z = 1.01, *P =* 0.31). Given these findings, we combined F1 cross directions in subsequent analyses.

**Fig. 6:**
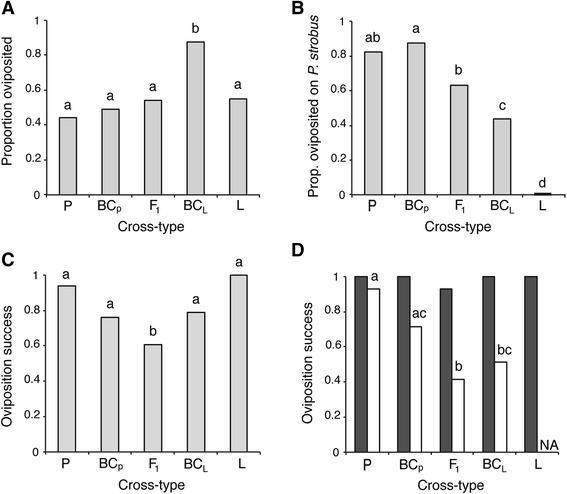
Oviposition preference and success depends on cross-type and host. **a** Proportion of females from each cross-type that laid eggs when placed within a host choice arena. Compared to other cross-types, BC_L_ females were more willing to oviposit when placed in a host choice arena. **b** Proportion of egg-laying females that chose *P. strobus*. Preference for *P. strobus* declined as the proportion of *N. lecontei* alleles increased. **c** Oviposition success (proportion of females with at least one hatching egg) was significantly lower for F1 females, indicating that there is post-zygotic isolation. **d** Oviposition success was lower on *P. strobus* (*white bars*) than on *P. banksiana* (*gray bars*) (*P* < 0.05); this host-dependent reduction in fitness is consistent with extrinsic postzygotic isolation. Compared to P and BC_P_ females, F1 and BC_L_ females had lower oviposition success on *P. strobus*. However, the host-by-cross-type interaction was not significant (*P* > 0.05). Oviposition success data are not available for “L” females on *P. strobus* because no L females chose *P. strobus* in this experiment (“NA”). In all panels, statistical significance at *P* < 0.05 is indicated by differing letters (see Table 1). In (**d**), letters refer to oviposition success on *P. strobus* only (no differences were observed on *P. banksiana*). Cross-type abbreviations are as indicated in Fig. 2

Females from the different cross-types differed significantly in their willingness to oviposit (χ^2^_4_ = 46.37, *P* <0.001, Fig. 6a). In particular, BC_L_ females oviposited significantly more often than all other types of females (Table 1). The cross-types also differed in their preference for *P. strobus*, with preference for this host declining as the individuals became more genetically different from *N. pinetum* (χ^2^_4_ = 82.40, *P* <0.001, Fig. 6b). None of the *N. lecontei* in our cross oviposited on *P. strobus*. The only cross-types that did not differ significantly in their *P. strobus* preference were P vs. BC_P_ and P vs. F1 (Table 1).

**Table 1:** Post hoc tests (Z-tests) for interspecific crosses

	Oviposition willingness		Oviposition preference		Oviposition success		Success on *P. strobus*		Success on *P. banksiana*	
Comparison	Z-score	P	Z-score	P	Z-score	P	Z-score	P	Z-score	P
*N. pinetum* vs. Bc_p_	0.67	0.50	0.16	0.87	−1.62	0.10	−1.57	0.12	*	*
*N. pinetum* vs. F1 Hybrids	1.29	0.12	−1.95	0.051	−4.14	**<1 × 10** ^**−04**^	−3.11	**1.88 × 10** ^**−03**^	0.48	0.63
*N. pinetum* vs. BC_L_	5.88	**<1 × 10** ^**−04**^	−3.60	**3 × 10** ^**−04**^	−1.55	0.12	−2.83	**4.66 × 10** ^**−03**^	*	*
*N. pinetum* vs. *N, lecontei*	0.76	0.45	−4.7	**<1 × 10** ^**−04**^	0.72	0.47	*NA*	*NA*	*	*
BC_P_ vs. F1 Hybrid	0.56	0.57	−2.19	**0.03**	−3.16	**1.58 × 10** ^**−03**^	−1.98	**0.047**	0.48	0.63
BC_P_ vs. BC_L_	5.15	**<1 × 10** ^**−04**^	−3.92	**<1 × 10** ^**−04**^	0.29	0.77	−1.60	0.11	*	*
BC_P_ vs. *N. lecontei*	0.27	0.79	−4.95	**<1 × 10** ^**−04**^	1.62	0.1	*NA*	*NA*	*	*
F1 Hybrid vs BC_L_	4.87	**<1 × 10** ^**−04**^	−2.01	**0.04**	4.6	**<1 × 10** ^**−04**^	0.59	0.56	1.81	0.07
F1 Hybrid vs. *N. lecontei*	−0.12	0.9	−3.62	**3 × 10** ^**−04**^	3.49	**4.8 × 10** ^**−04**^	*NA*	*NA*	0.82	0.41
BC_L_ vs. *N. lecontei*	−3.62	**3 × 10** ^**−04**^	−2.73	**6.34 × 10** ^**−03**^	1.54	0.12	*NA*	*NA*	*	*

*NA* samples unavailable

* = Z-score undefined when both proportions are 1. **Bold** represents significance at *P* < 0.05

The cross-types also differed in their oviposition success (χ^2^_4_ *=* 13.03, *P* = 0.011, Fig. 6c), and the F1 females had significantly lower hatching success than any of the other cross-types (Table 1). When oviposition host and an interaction between host and cross-type were added, cross-type remained significant (χ^2^_4_ = 14.92, *P =* 0.0049, Fig. 6d). Additionally, there was a significant effect of host on oviposition success (χ^2^_1_ = 44.43, *P* <0.001): across all cross-types, females that chose *P. strobus* had lower hatching success than females that chose *P. banksiana* (Fig. 6d). Also, although none of the *N. lecontei* females involved in the cross chose *P. strobus*, four of the *N. lecontei* females from our multi-population preference experiment did chose *P. strobus* (Fig. 3b). Notably, all four of these females experienced complete hatching failure (Additional file 1: Figure S3). Although both cross-type and oviposition host significantly impacted hatching success, the interaction between them was not significant (χ^2^_3_ = 2.37, *P =* 0.50). We also found that rearing host did not affect the oviposition success of backcross females (χ^2^_1_ = 0.22, *P* = 0.64).

### Impact of oviposition traits on BC_L_ oviposition success

**Fig. 7:**
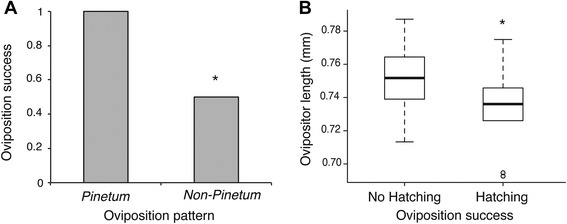
BC_L_ females with *pinetum*-like oviposition traits have higher oviposition success on *P. strobus*. **a** Oviposition success (proportion of females with at least one hatching egg) was higher for BC_L_ females that had a *pinetum*-like oviposition pattern (<3 eggs per needle) compared to females that lacked this pattern (>3 eggs per needle) (*P <*0.05). **b** Females that laid successfully had shorter ovipositors than those that did not (*P* < 0.05)

BC_L_ females that had a *pinetum*-like oviposition pattern were significantly more likely to have eggs that hatched on *P. strobus* than if they deviated from this pattern (χ^2^_1_ = 3.85, *P* =0.0498, Fig. 7a). Also, BC_L_ females that successfully oviposited on *P. strobus* had significantly shorter ovipositors than unsuccessful females (χ^2^_1_ = 9.50, *P =* 0.0021, Fig. 7b). In contrast, successful and unsuccessful females did not differ in ovipositor width (χ^2^_1_ = 0.019, *P* = 0.89) or ovipositor shape (F_1, 17_ = 1.16, *P* = 0.24).

In BC_L_ females, host choice (*P. strobus* vs. *P. banksiana*) did not correlate with oviposition pattern (χ^2^_1_ = 0.14, *P* = 0.70), ovipositor length (F_1, 38_ = 1.81, *P* = 0.19), ovipositor width (F_1, 38_ = 0.0056, *P* = 0.94), or ovipositor shape (F_1, 38_ = 1.86, *P* = 0.22). Finally, oviposition pattern was unrelated to ovipositor length (F_2,17_ = 0.20, *P* = 0.82), ovipositor width (F_2, 17_ = 0.024, *P* = 0.98), or ovipositor shape (F_2, 17_ = 1.10, *P =* 0.35). Together, these results imply that host preference, oviposition pattern, and ovipositor morphology are genetically independent traits.

## Discussion

Empirical data from diverse taxa indicate that ecological speciation is common in nature [7–10], and that changes in host use frequently initiate ecological speciation in plant-feeding insects [27, 29]. However, the contributions of specific divergent traits to EPI are unknown in most systems. In this study, we evaluated evidence of oviposition traits generating extrinsic postzygotic isolation between a pair of *Neodiprion* sawfly species that specialize on different pines. We found compelling evidence of EPI stemming from maladaptive combinations of oviposition traits. Here, we discuss the limitations, as well as broader implications of our work for ecological specialization and speciation in plant-feeding insects and future research directions in this promising empirical system.

Although all sawflies in the genus *Neodiprion* feed on host plants in the family Pinaceae (mostly in the genus *Pinus*), different sawfly species tend to specialize on different pine hosts [34, 45]. Previous analyses at both the inter- and intraspecific levels indicate that changes in host use are associated with population differentiation and speciation in this genus [25, 35]. In this study, we investigated a potential causal relationship between adaptation to different hosts and reproductive isolation. In particular, we hypothesized that maladaptive combinations of divergent oviposition traits give rise to extrinsic postzygotic isolation between *Neodiprion* species*.* Consistent with this hypothesis, we found that sister species *N. pinetum* (a thin-needled specialist) and *N. lecontei* (occurs on thicker-needled hosts) differed in multiple behavioral and morphological traits related to oviposition (Figs. 3, 4 and 5). In terms of behavior, *N. pinetum* females preferred *P. strobus* (white pine), laid a small number of widely spaced eggs on each needle, and never cut resin-draining preslits. In contrast, *N. lecontei* females generally avoided *P. strobus*, laid many closely spaced eggs per needle, and almost always cut preslits. In terms of morphology, *N. pinetum* females had smaller, straighter ovipositors than *N. lecontei* females. Together, *N. pinetum* traits likely enable females to insert eggs into *P. strobus* without damaging the thin needles to the point that they dry out and the eggs die, while *N. lecontei* traits should better equip females to circumvent host defenses and prevent eggs from being overwhelmed by resin.

Although *N. lecontei* and *N. pinetum* appear to be specialized to oviposit on different hosts, they do hybridize in nature (*personal observation*, [35]), indicating that premating barriers are incomplete. Nevertheless, the strong genetic, behavioral, and morphological differentiation between these two sympatric species ([35]; Figs. 3, 4 and 5) suggests that there are postzygotic barriers to gene exchange. Consistent with this prediction, we found that F1 females had reduced oviposition success relative to the two parental species (Fig. 6c). For these females, there were two potential sources of oviposition failure: botched oviposition (which would be host-dependent and therefore extrinsic in nature) and egg inviability (which could stem from intrinsic genetic incompatibilities or from extrinsic egg-host interactions). Our observation that hybrid females had reduced oviposition success only when they chose *P. strobus* suggests that postzygotic isolation between *N. lecontei* and *N. pinetum* is largely attributable to extrinsic, rather than intrinsic, factors. By contrast, oviposition success of hybrid females on the more “benign” *P. banksiana* seedlings was indistinguishable from oviposition success of pure *N. lecontei* and *N. pinetum* females. Although this finding is consistent with EPI, an alternative explanation for these results is that intrinsic genetic incompatibilities between the species are more pronounced in the *P. strobus* environment [7, 13, 18]. One way to control for intrinsic genetic incompatibilities is to compare the fitness of both backcross types in both parental environments [13]. Using this method, we found that BC_P_ females had high oviposition success on both hosts, while BC_L_ females had high oviposition success on *P. banksiana* only (Fig. 6d). While patterns observed in BC_L_ females are consistent with EPI, patterns observed in BC_p_ are not.

While seemingly at odds with predictions under EPI, our observation that BC_P_ females had high oviposition success on both hosts could be attributable to our experimental design. There are two main sources of experimental error that could have precluded us from detecting reduced hatch success on *P. banksiana*. First, by scoring oviposition success as a binary trait (hatch or no hatch), we lumped together females with a wide range of hatching success (from <10 to 100%). Failure to account for variation in non-zero hatch success would have reduced our power to detect all but the most extreme differences in oviposition success. In other words, while *lecontei*-like oviposition traits led to a complete failure on *P. strobus* consistently enough that we could detect it with our crude measure of success, we had little power to detect subtler reductions in hatching success.

The second potential source of experimental error in our assessment of EPI is that we used pine seedlings in lieu of larger trees, which we could not accommodate in our growth rooms. However, as our needle width data indicate (Additional file 1: Figure S2), the pine seedlings we used did not fully recapitulate differences in the host age classes that are typically selected by ovipositing *N. lecontei* and *N. pinetum* females in the wild [53, 57]. In particular, the needles of our *P. banksiana* seedlings were considerably thinner than needles from older trees. Moreover, resin content tends to increase as pine trees age [58]. Thus, while the *P. strobus* seedlings we used replicated the challenge of laying eggs on a thin needled-host, the *P. banksiana* seedlings did not replicate the challenge of laying on a thick, resinous needle.

Despite these possible experimental artifacts, we do have an additional line of direct evidence supporting the existence of EPI due to oviposition traits: on *P. strobus*, BC_L_ females with *lecontei*-like oviposition traits (ovipositor morphology and egg-laying behavior) had reduced oviposition success compared to BC_L_ females with *pinetum*-like oviposition traits (Fig. 7). Because all BC_L_ females share the same genetic makeup (i.e., same proportion of *N. lecontei* and *N. pinetum* alleles), these differences cannot be explained by intrinsic genetic incompatibilities. Taken together, our cross data indicate that maladaptive combinations of oviposition preference and oviposition traits in hybrids generate EPI between *N. lecontei* and *N. pinetum.* Intriguingly, maladaptive combinations of preference and performance traits have been reported in several other insect taxa [20, 29, 59, 60], suggesting that this might be a widespread cause of reduced hybrid fitness.

Our analysis of traits in BC females also demonstrates how examination of specific traits in hybrid individuals can be used as an alternative to the “modify-parental-phenotype” test of EPI that has been proposed, but never utilized [7]. In our case, modifying parental phenotypes was not an option because our focal phenotypes were either behavioral (host preference, oviposition pattern) or involved a delicate morphological structure (ovipositor) that we could not alter readily—we suspect that the same is probably true of many organisms in which one might want to investigate EPI. However, as we have shown here, genetic crosses can serve a similar function as parental modification. In particular, by generating recombination among loci underlying ecologically relevant traits and assessing fitness in recombinant individuals, we could begin to tease apart how individual traits and interactions between them contribute to reduced fitness of hybrids in parental environments. To date, we know of only one other study that has taken advantage of trait variation in hybrids to make inferences regarding EPI in plant-feeding insects: McBride and Singer’s [20] study of EPI in *Euphydryas* butterflies (see also [18] for an example in Caribbean pupfishes). In their study, McBride and Singer reared F1 hybrids between allopatric, host-specialized populations on both parental hosts and, for four behavioral traits, found that trait intermediacy in the hybrids reduced their fitness on both hosts.

To date, numerous studies—many of which focused on plant-feeding insects—have reported evidence of EPI (see [8, 28, 29]). While only a handful of these have employed a more rigorous approach (e.g., reciprocal backcross or trait-focused studies) that controls for genetic incompatibilities [20, 30–33], the emerging picture from this body of work is that EPI frequently accompanies ecological speciation. However, in only a handful of cases have the traits underling EPI been identified [18, 20]. Importantly, although EPI is a direct consequence of adaptive divergence, adaptive divergence does not always produce EPI. For example, if intermediate trait values do not impact fitness in parental environments or if an intermediate environment is available in nature, hybrids will not experience ecologically based reductions in fitness [15]. As more traits are explicitly tested for their role in EPI, we can begin to ask more specific questions about its mechanistic basis, such as: which traits (behavior, physiology, morphology) and which aspects of ecology (reproduction, food acquisition and processing, parasitism) are most likely to produce EPI?

Based on their findings in *Euphydryas* butterflies, McBride and Singer [20] proposed that behavioral traits—especially niche preferences—might be especially important drivers of EPI. In support of this argument they provided two additional examples. First, two European blackcap populations that migrate in opposite directions to their wintering grounds produce hybrids with a tendency to migrate in an intermediate and maladaptive direction [61]. Second, hybrids between apple- and hawthorn host races of *Rhagoletis pomonella* have a tendency to avoid both parental hosts, making it difficult for them to locate suitable oviposition sites [62, 63]. By contrast, our hybrids did not exhibit a reduction in willingness to oviposit (in fact, for reasons that are currently unclear to us, BC_L_ seemed more willing to oviposit than other cross types; Fig. 6), indicating that “host confusion” is not contributing to EPI in this system. Nevertheless, our data are consistent with the overall importance of behavioral traits (in our case, host preference and oviposition pattern) in driving EPI.

Additionally, similar to our finding that oviposition traits contribute to EPI in *Neodiprion*, three of the four traits implicated in reduced hybrid fitness in *Euphydryas* butterflies were related to oviposition. Experimental and natural history work in additional *Neodiprion* species suggest that this phenomenon might be widespread in pine sawflies as well. Specifically, for several different *Neodiprion* species, there are published observations of egg mortality caused by either needle desiccation or drowning in resin, and these outcomes are often associated with needle thickness, needle resin content, and female oviposition pattern (e.g., number of eggs per needle, presence of preslit, depth of egg slits and preslits) [41–44, 64–67]. Together, these observations suggest that oviposition traits are under strong selection both within and between *Neodiprion* species. Intriguingly, host preference, ovipositor morphology, and oviposition pattern are also among the most variable traits in the genus and are often useful in species identification [45, 68, 69]. If host-related selection has shaped this variation, inter- and intraspecific variation in host preference should correlate with variation in other oviposition traits; this prediction could be tested using a comparative approach.

Beyond *Neodiprion*, oviposition-related traits—which include traits related to finding and choosing a host, selecting a site within the host for egg deposition, depositing eggs in specific patterns on or within the host tissue, defusing host defenses, ovipositor morphology, and egg morphology—could profoundly impact the fitness of any egg-laying phytophagous insect female and are therefore likely to be frequent targets of natural selection [70]. In support of this argument, numerous studies have reported host-associated differentiation in oviposition traits, including: clutch size in seed beetles [71], ovipositor morphology in yucca moths [72], ovipositor length in gall-inducing *Asphodylia* flies [73], ovipositor length in fig wasps [74], ovipositor size in *Plateumaris* leaf beetles [75], clutch size and oviposition site in butterflies [76], and multiple morphological and behavioral traits in pine sawflies (this study). However, in the context of traits driving ecological specialization and speciation in plant-feeding insects, research has focused almost exclusively on female host preference and larval performance (i.e., growth and survival rates when feeding on a particular host plant). To understand the role of host specialization in phytophagous insect speciation, it is critical that we examine additional host-related traits.

## Conclusions

In this study, we have demonstrated that oviposition traits contribute to EPI between *N. lecontei* and *N. pinetum*. While these observations are consistent with ecological speciation*,* the evidence is not yet iron-clad and many important questions remain. First, while we focused here on oviposition traits, other traits—such as larval performance—could also contribute to EPI. In future work, we hope to quantify the impact of individual traits—and the interaction between them—on host-dependent reductions in hybrid fitness. Second, while we have focused here on EPI, there are other sources of reproductive isolation between these species (*personal observation*). To evaluate the contribution of EPI to total isolation, we must quantify the strength of EPI relative to other reproductive barriers [77, 78]. Finally, understanding how divergent traits give rise to RI requires that we identify the genetic mechanisms (i.e., linkage or pleiotropy) linking them [7, 8]. As we have demonstrated here, these species are interfertile in the lab; thus, a QTL mapping approach is feasible in this system. Additionally, identification of causal loci—which is required if we are to distinguish between pleiotropy and linkage—will be facilitated by the availability of annotated genome assemblies for *N. lecontei* [79] and *N. pinetum* (in progress).

While a long-term goal is to identify all host-related traits under selection, all reproductive barriers, and their underlying genes in *N. lecontei* and *N. pinetum*, these efforts will provide a single snapshot at one time point in speciation. Because these species have been diverging for up to several million years [25, 80], they have had time to accumulate many differences and barriers to reproduction, which will make it difficult to determine which reproductive barriers arose first. To get at this question, we can examine other *Neodiprion* species and populations at different stages along the “speciation continuum” [81]. For example, there is evidence of host-associated differentiation in at least two *Neodiprion* species (*Neodiprion lecontei;* [35]; *Neodiprion abietis,* [42, 82]), and possibly other *Neodiprion* species as well. Although much work remains, extensive natural history data, experimental tractability, and growing genomic resources make *Neodiprion* an exceptionally rich system for addressing many long-standing questions regarding the evolution of host specialization and its role in generating the staggering diversity of phytophagous insects.

## Additional file

Additional file 1: Table S1. Collection locations and number of females from each population populations used in parental phenotyping experiments. **Table S2.** Collection locations for mature pine needles. **Table S3.** Sample sizes for interspecific crosses. **Table S4.** Tukeys HSD for mature needle widths. **Table S5.** Tukeys HSD post hoc test for *P. strobus* and *P. banksiana* needle widths. **Figure S1.** Host-dependent fitness ranking predictions under extrinsic postzygotic isolation. **Figure S2.** Seedling needles partially recapitulate differences between mature *P. banksiana* and *P. strobus*. **Figure S3.** Oviposition success of *N. lecontei* and *N. pinetum* on *P. banksiana and P. strobus.* (DOCX 211 kb)

## Abbreviations

EBN: Egg bearing needle
EPI: Extrinsic postzygotic isolation
RI: Reproductive isolation
