# Supplementary material for: Oviposition traits generate extrinsic postzygotic isolation between two pine sawfly species
Source: BMC Evol Biol. 2017 Jan 19;17:26. doi: 10.1186/s12862-017-0872-8 (PMC5248504; doi:10.1186/s12862-017-0872-8)
Supplement: Additional file 1: Table S1. — Collection locations and number of females from each population populations used in parental phenotyping experiments. Table S2. Collection locations for mature pine needles. Table S3. Sample sizes for interspecific crosses. Table S4. Tukeys HSD for mature needle widths. Table S5. Tukeys HSD post hoc test for P. strobus and P. banksiana needle widths. Figure S1. Host-dependent fitness ranking predictions under extrinsic postzygotic isolation. Figure S2. Seedling needles partially recapitulate differences between mature P. banksiana and P. strobus. Figure S3. Oviposition success of N. lecontei and N. pinetum on P. banksiana and P. strobus. (DOCX 211 kb) [file 12862_2017_872_MOESM1_ESM.docx]

**Additional file**

Table S1. Collection locations and number of females from each population populations used in parental phenotyping experiments.

| Species | City, State | Latitude | Longitude | Collection Host | Oviposition Willingness | Oviposition  Preference | Eggs Per Needle | Preslit | Egg Spacing | Ovipositor Morphology |
| --- | --- | --- | --- | --- | --- | --- | --- | --- | --- | --- |
| ***N. pinetum*** | **Crossville, TN** | **35.980** | **-85.015** | ***P. strobus*** | 43 | 23 | 6 | 0 | 5 | 5 |
| *N. pinetum* | Lexington, KY | 38.042 | -84.442 | *P. strobus* | 5 | 5 | 0 | 0 | 0 | 5 |
| *N. pinetum* | Lexington, KY | 38.003 | -84.525 | *P. strobus* | 2 | 1 | 1 | 1 | 1 | 0 |
| *N. pinetum* | Lexington, KY | 38.032 | -84.565 | *P. strobus* | 5 | 4 | 4 | 4 | 3 | 5 |
| *N. pinetum* | Lexington, KY | 37.971 | -84.498 | *P. strobus* | 1 | 1 | 1 | 1 | 1 | 5 |
| *N. pinetum* | Lexington, KY | 37.973 | -84.500 | *P. strobus* | 4 | 2 | 1 | 1 | 0 | 5 |
| *N. pinetum* | Georgetown, KY | 38.249 | -84.549 | *P. strobus* | 2 | 2 | 0 | 0 | 0 | 0 |
| *N. pinetum* | Florence, KY | 39.008 | -84.65 | *P. strobus* | 2 | 2 | 0 | 0 | 0 | 0 |
| Total |  |  |  |  | 64 | 40 | 13 | 7 | 10 | 25 |
| *N. lecontei* | Lexington, KY | 38.014 | -84.504 | *P. virginiana* | 25 | 16 | 16 | 14 | 10 | 5 |
| *N. lecontei* | Crossville, TN | 35.980 | -85.015 | *P. virginiana* | 6 | 3 | 3 | 3 | 2 | 5 |
| *N. lecontei* | Spooner, WI | 44.600 | -84.713 | *P. banksiana* | 6 | 2 | 2 | 2 | 2 | 0 |
| ***N. lecontei*** | **Lexington, KY** | **38.014** | **-84.504** | ***P. echinata*** | 44 | 31 | 0 | 0 | 0 | 5 |
| *N. lecontei* | Lexington, KY | 38.014 | -84.504 | *P. rigida* | 0 | 0 | 0 | 0 | 0 | 5 |
| *N. lecontei* | Goshen, KY | 38.402 | -85.586 | *P. echinata* | 0 | 0 | 0 | 0 | 0 | 5 |
| Total |  |  |  |  | 81 | 52 | 21 | 19 | 14 | 25 |

**Bold** indicates populations used in the interspecific crosses

Table S2. Collection locations for mature pine needles

| Pine Species | City, State | Latitude | Longitude |
| --- | --- | --- | --- |
| *P. banksiana* | Necedah, WI | 44.115 | -90.118 |
| *P. echinata* | Lexington, KY | 37.973 | -84.500 |
|  | London, KY | 37.071 | -84.211 |
| *P. resinosa* | Necedah, WI | 44.115 | -90.118 |
| *P. rigida* | Lexington, KY | 37.973 | -84.500 |
|  | Liberty, KY | 37.221 | -84.956 |
| *P. strobus* | Lexington, KY | 38.034 | -84.506 |
| *P. virginiana* | Lexington, KY | 37.973 | -84.500 |
|  | London, KY | 37.071 | -84.211 |

Table S3. Sample sizes for interspecific crosses

| Cross-type | Oviposition Willingness | Oviposition Preference | Oviposition Success | Success on *P. Strobus* | Success on *P. banksiana* |
| --- | --- | --- | --- | --- | --- |
| *N. pinetum* | 54 | 23 | 18 | 15 | 3 |
| BC_P_ | 53 | 26 | 25 | 22 | 3 |
| F1 Hybrid | 72 | 39 | 37 | 25 | 12 |
| BC_L_ | 97 | 85 | 80 | 35 | 45 |
| *N. lecontei* | 19 | 10 | 9 | 0 | 9 |

Table S4. Tukeys HSD for mature needle widths

| Comparison | Difference | Lower 95% CI | Upper 95% CI | P-value |
| --- | --- | --- | --- | --- |
| *P. strobus* vs *P. echinata* | -0.34 | -0.51 | -0.18 | **1.8x10^-6^** |
| *P. strobus* vs. *P. resinosa* | -0.51 | -0.68 | -0.34 | **<1x10^-7^** |
| *P. strobus* vs. *P. virginiana* | -0.45 | -0.62 | -0.28 | **<1x10^-7^** |
| *P. strobus* vs. *P. banksiana* | -0.84 | -1.00 | -0.67 | **<1x10^-7^** |
| *P. strobus* vs. *P. rigida* | -0.93 | -1.10 | -0.76 | **<1x10^-7^** |
| *P. echinata* vs. *P. resinosa* | -0.17 | -0.33 | -0.00 | **0.047** |
| *P. echinata* vs. *P. virginiana* | 0.11 | -0.06 | 0.27 | 0.40 |
| *P. echinata* vs. *P. banksiana* | -0.49 | -0.66 | -0.33 | **<1x10^-7^** |
| *P. echinata* vs. *P. rigida* | -.06 | -0.75 | -0.42 | **<1x10^-7^** |
| *P. resinosa* vs. *P. virginiana* | -0.06 | -0.23 | 0.11 | 0.89 |
| *P. resinosa* vs. *P. banksiana* | -0.33 | -0.49 | -0.16 | **5.3x10^-6^** |
| *P. resinosa* vs. *P. rigida* | -0.42 | -0.58 | -0.25 | **<1x10^-7^** |
| *P. virginiana* vs. *P. banksiana* | -0.39 | -0.55 | -0.22 | **1x10^-7^** |
| *P. virginiana* vs. *P. rigida* | -0.48 | -0.64 | -0.31 | **<1x10^-7^** |
| *P. banksiana* vs *P. rigida* | -0.09 | -0.07 | 0.26 | 0.58 |

Table S5. Tukeys HSD post hoc test for *P. strobus* and *P. banksiana* needle widths

| Comparison | Difference | Lower 95% CI | Upper 95% CI | P-value |
| --- | --- | --- | --- | --- |
| Mature *P. strobus* vs. Mature *P. banksiana* | -0.84 | -0.94 | -0.73 | **<1x10^-7^** |
| *P. banksiana* Seedling vs. Mature *P. banksiana* | -0.39 | -0.50 | -0.29 | **<1x10^-7^** |
| *P. strobus* Seedling vs. Mature *P. banksiana* | -0.75 | -0.86 | -0.64 | **<1x10^-7^** |
| *P. banksiana* Seedling vs. Mature *P. strobus* | 0.44 | 0.34 | 0.55 | **<1x10^-7^** |
| *P. strobus* Seedling vs. Mature *P. strobus* | 0.09 | -0.02 | 0.19 | 0.15 |
| *P. strobus* Seedling vs. *P. banksiana* Seedling | -0.36 | -0.46 | -0.25 | **<1x10^-7^** |

**
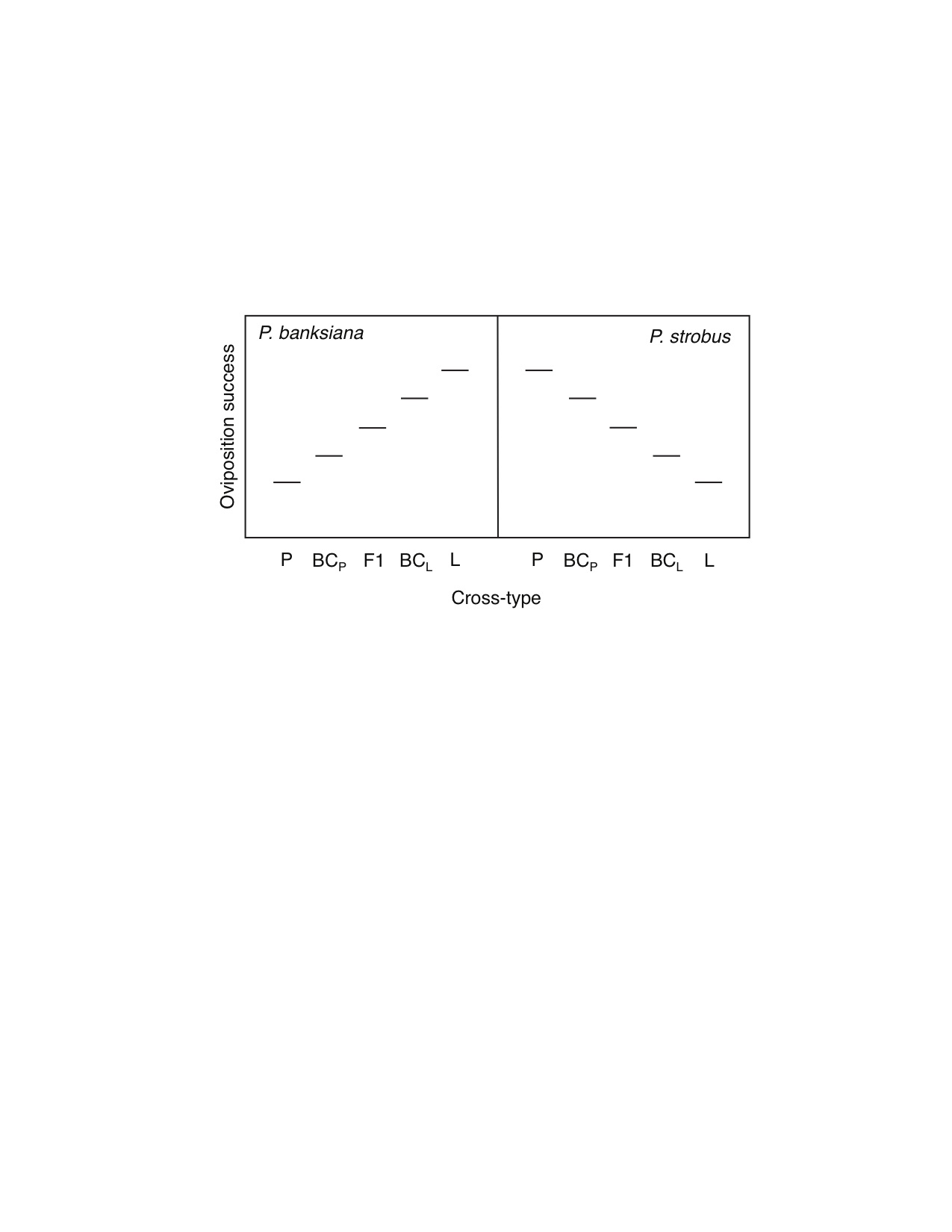
**

Figure S1. Host-dependent fitness ranking predictions under extrinsic postzygotic isolation. Cross-type is indicated as in Fig. 2 (P= *N. pinetum*, BC_P_ = *N. pinetum* backcross, F1= F_1_ hybrids. BC_L_= *N. lecontei* backcross, L= *N. lecontei).*


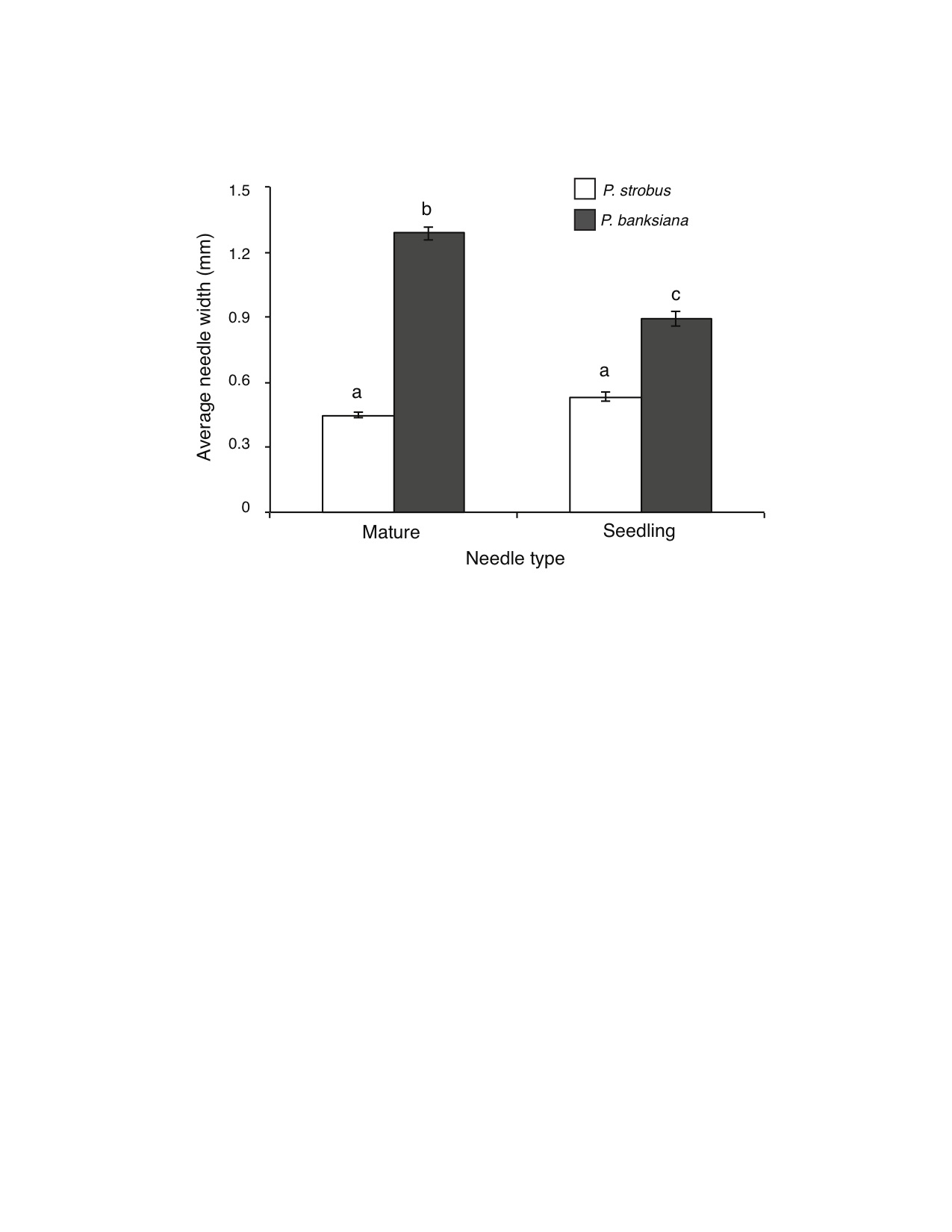


Figure S2. Seedling needles partially recapitulate differences between mature *P. banksiana* and *P. strobus*. Mean widths (+/- SEM) for needles taken from seedlings and mature pines (*P. banksiana* and *P. strobus*)*.* Although *P. banksiana* needles are always thicker than *P. strobus* needles, *P. banksiana* seedlings have thinner needles than mature *P. banksiana* trees. Statistical significance at *P* < 0.05 is indicated by differing letters (Table S5)**.**


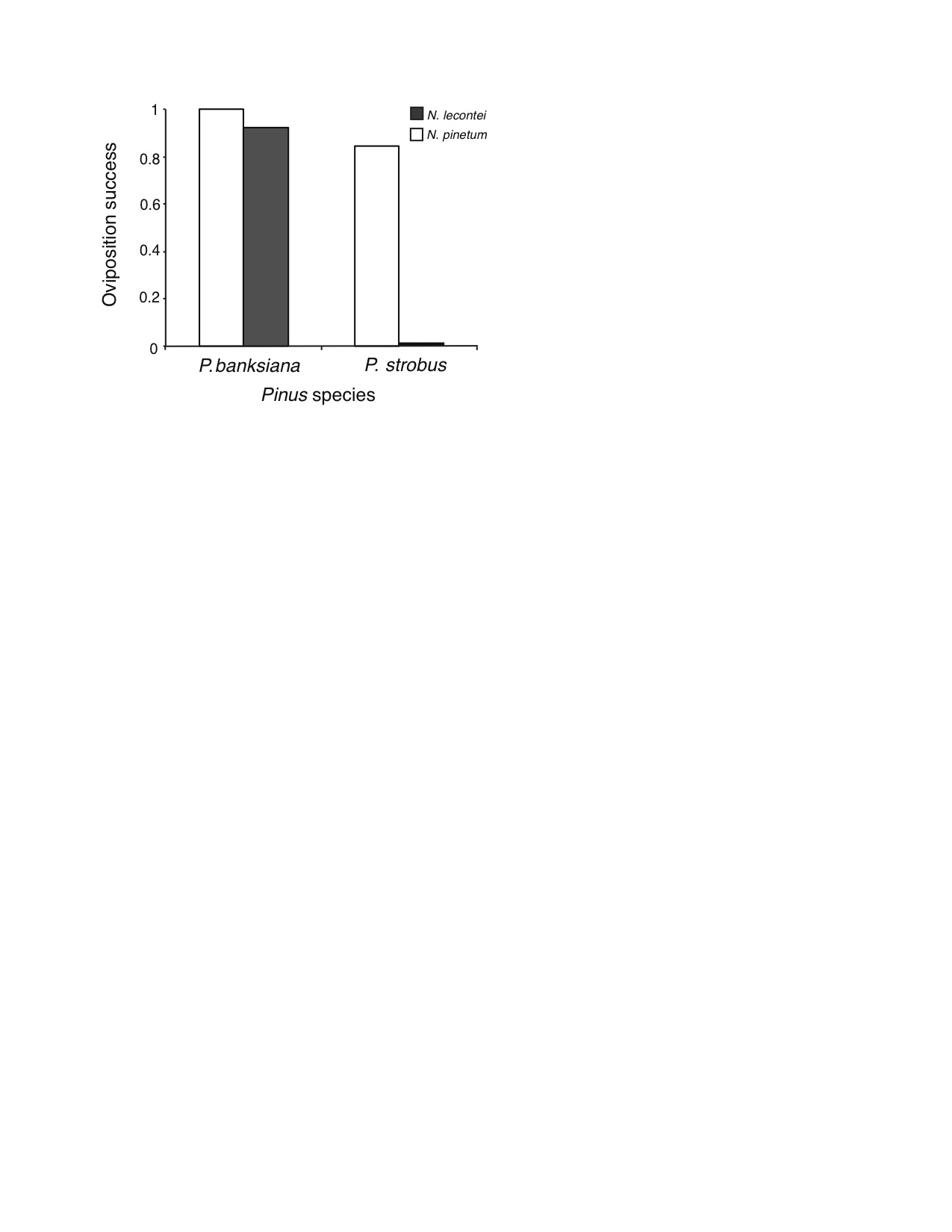


Figure S3. Oviposition success of *N. lecontei* and *N. pinetum* on *P. banksiana* *and P. strobus.* On *P. strobus*, *N. lecontei* has complete failure to hatch.
